# Zirconium-89 labelled rituximab PET-CT imaging of Graves’ orbitopathy

**DOI:** 10.1007/s00259-019-04599-8

**Published:** 2019-11-18

**Authors:** Bart de Keizer, Kamil G. Laban, Rachel Kalmann

**Affiliations:** 1 Department of Radiology and Nuclear Medicine, University Medical Center Utrecht, University Utrecht, Heidelberglaan 100, 3584 CX Utrecht, The Netherlands; 2 Department of Ophthalmology, University Medical Center Utrecht, University Utrecht, Utrecht, The Netherlands; 3 Laboratory of Translational Immunology, University Medical Center Utrecht, University Utrecht, Utrecht, The Netherlands

## Zirconium-89 labelled rituximab PET-CT imaging of Graves’ orbitopathy

**Figure 1:**
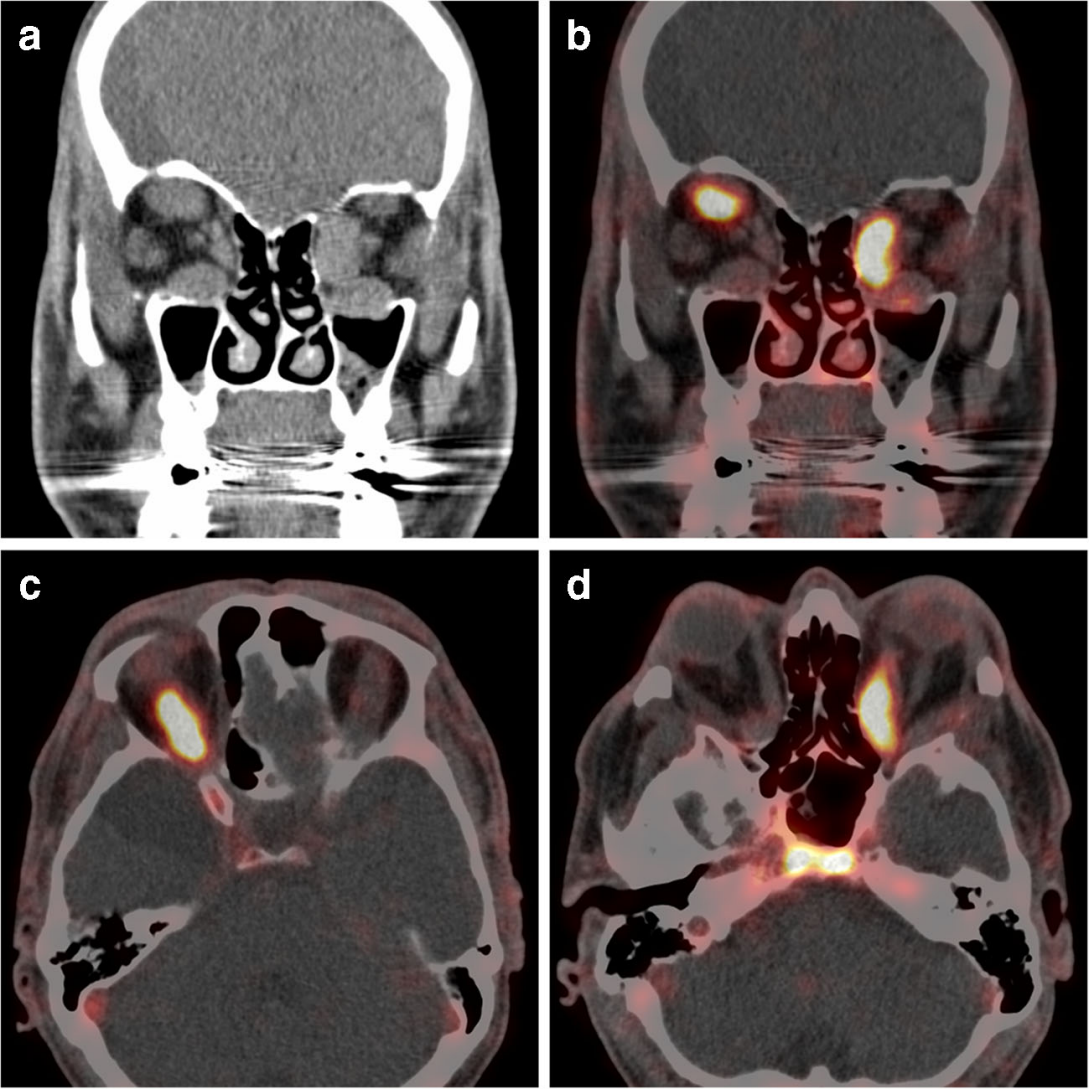
Figure 1

Graves’ orbitopathy (GO) is the main extrathyroidal manifestation of Graves’ disease. A proportion of patients have moderate to severe orbital inflammation, with corneal ulceration, intense pain or even compressive optic neuropathy [1]. High-dose glucocorticoids (GCs) are the first-line treatment in these patients. When high-dose GCs fail to reduce the inflammation, shared decision-making is recommended for selecting a second-line treatment. Options for treatment include a second course of intravenous GCs, oral GCs combined with orbital radiotherapy, rituximab or watchful waiting [2]. Rituximab treatment is not yet approved for clinical use in GO and roughly 50% do not have significant improvement 1 year after treatment [3]. In rheumatoid arthritis, zirconium-89-labelled rituximab (^89^Zr-rituximab) PET-CT shows promising clinical value with higher rates of response to therapy in patients with higher ^89^Zr-rituximab uptake in responders than in non-responders [4]. ^89^Zr-rituximab PET scanning is approved by Dutch authorities to select patients for rituximab treatment and is used in our hospital to select patients with orbital inflammatory disease (including GO) that might benefit from rituximab treatment*.* In a recent retrospective study, we showed that of 4 patients with intense ^89^Zr-rituximab uptake in orbital inflammatory disease, 3 patients responded well to rituximab treatment [5]. Here, we present a patient with GO refractory to intravenous GCs. PET-CT performed 3 days after 74 MBq ^89^Zr-rituximab showed high uptake in orbital musculature. ^89^Zr-rituximab binding more than in normal bone marrow and comparable to binding in normal lymph nodes was observed in thickened medial rectus muscle of the left eye (SUVmax 5.9) and the superior rectus muscle of the right eye (SUVmax 5.2) (Figure 1 A coronal CT reconstruction, B coronal PET-CT reconstruction, C axial PET-CT reconstruction of right superior rectus muscle and D axial PET-CT reconstruction of left medial rectus muscle). Because of high ^89^Zr-rituximab uptake, rituximab treatment was initiated.
